# Millennial-scale microbiome analysis reveals ancient antimicrobial resistance conserved despite modern selection pressures

**DOI:** 10.1186/s40793-024-00652-8

**Published:** 2024-12-18

**Authors:** Sankaranarayanan Gomathinayagam, Swathi Kanagalingam, Srimathi Chandrasekaran, Thirumoorthy Krishnan, Gothandam Kodiveri Muthukaliannan

**Affiliations:** 1 Department of Biotechnology, School of Biosciences and Technology, Vellore Institute of Technology, Vellore, Tamil Nadu 632014 India; 2 Department of Information Security, School of Computer Science, Vellore Institute of Technology, Vellore, Tamil Nadu India; 3 Department of Chemistry, School of Advanced Sciences, Vellore Institute of Technology, Vellore, Tamil Nadu 632 014 India

**Keywords:** Ancient permafrost microbiome, Antimicrobial resistance, Bacteriophages, Selection pressure, dN/dS ratio

## Abstract

**Background:**

Antimicrobial resistance presents a formidable challenge, yet its existence predates the introduction of antibiotics. Our study delves into the presence of antimicrobial resistance genes (ARGs) in ancient permafrost microbiomes, comparing them with contemporary soil and pristine environments. Majority of the samples are from regions around Beringia, encompassing parts of Russia and Alaska, with only one sample originating from the Tien Shan Mountain range in Kyrgyzstan.

**Results:**

From over 2.3 tera basepairs of raw metagenomic data, retrieved from samples ranging in age from approximately 7,000 years to 1.1 million years, we assembled about 1.3 billion metagenomic contigs and explored the prevalence of ARGs within them. Our findings reveal a diverse array of ARGs in ancient microbiomes, akin to contemporary counterparts. On average, we identified 2 ARGs per rRNA gene in ancient samples. *Actinomycetota*, *Bacillota*, and several thermophiles were prominent carriers of ARGs in Chukochi and Kamchatkan samples. Conversely, ancient permafrost from the Tien Shan Mountain range exhibited no Thermophiles or *Actinomycetota* carrying ARGs. Both ancient and contemporary microbiomes showcased numerous divergent ARGs, majority of which have identity between 40 and 60% to genes in antibiotic resistance gene databases. To study the selection pressure on ARGs, we performed dN/dS analysis specifically on antibiotic inactivation-type ARGs, which exhibited purifying selection compared to contemporary genes.

**Conclusion:**

Antibiotic resistance has existed throughout microbial evolution and will likely persist, as microbes have the capacity to develop and retain resistance genes through evolutionary processes. The classes of antimicrobial resistance genes profiled and the function of antibiotic-inactivating enzymes from ancient permafrost microbiomes do not seem to be very different from the genes found in the antibiotic era. Additionally, we retrieved 359 putative complete viruses from ancient microbiomes and none of them harboured any ARGs.

**Supplementary Information:**

The online version contains supplementary material available at 10.1186/s40793-024-00652-8.

## Introduction

Microorganisms, as some of the oldest extant organisms, have endured myriad challenging environments over eons, predating significant events such as the Great Oxygenation Event and the subsequent oxygenated world. They have demonstrated remarkable resilience and adaptability, thriving for billions of years. Throughout their extensive history, microorganisms have encountered and adapted to numerous toxic environments, necessitating the development of mechanisms to mitigate such challenges. For instance, the transition to an oxygenated environment posed a threat to anaerobic microorganisms. Those microorganisms that evolved antioxidant mechanisms and other defensive strategies against environmental toxins were more likely to survive and undergo selection [1, 2]. 

The phenomenon of antimicrobial resistance likely originated alongside the production of antimicrobials by microorganisms. Evidence suggests that antimicrobial resistance existed within microbial populations even before the discovery and widespread use of antibiotics [3]. This antimicrobial synthesis and development of resistance is thought to have arisen as a result of competition within their ecological niches, illustrating the ancient and ongoing arms race between microorganisms and antimicrobial substances. But it would be interesting to investigate what the scenario of the antimicrobial resistome must have been in the microbial environment before the anthropocene, in contrast to the age of extensive antibiotic use.

There are few historical collections of bacterial cultures dating back to the time antibiotics were discovered, such as the collection assembled by E.G.D. Murray spanning the period from 1917 to 1954. This collection is known to still be maintained at the National Collection of Type Cultures, Public Health England. Collections like these would provide valuable insights into microbes from the time of clinical introduction of antibiotics [4].

Furthermore, studies examining historical microbiomes, such as those derived from dental calculus of ancient humans and animals, coprolites, mummified bones etc., offer another window into the past. In a previous study, we investigated antimicrobial resistance genes in Neanderthal dental calculus and coprolite microbiomes. However, we encountered challenges related to post-mortem DNA damage, which impeded the retrieval of complete antimicrobial resistance genes (ARGs) from these ancient microbiomes [5]. Additionally, contamination from soil and other environmental sources posed further obstacles to the accurate detection of ancient ARGs distinct from their modern counterparts. Moreover, the authenticity of the identified putative ARGs could not be confirmed through cytosine deamination (typical characteristic of ancient DNA fragment) at the ends of the reads corresponding to the respective ARGs.

Recent studies have highlighted the presence of ARGs in pristine cryospheric ecosystems, including glaciers, glacier-fed streams, and Antarctic soils, shedding light on the natural and anthropogenic factors shaping the resistome in these remote environments. Research has shown that even in relatively isolated habitats, such as glacier ice sheets and glacier-fed streams, ARGs are prevalent, often linked to the intrinsic resistomes of microbial communities or human activities. For instance, a study revealed that culturable antibiotic-resistant bacteria are significantly higher in areas of increased human impact, such as recreational sites near glaciers, demonstrating the influence of anthropogenic activities on environmental resistomes [6]. 

In glacier-fed streams, the resistomes of epilithic biofilms include ARGs with abundant mechanisms for resistance to beta-lactams, aminoglycosides, and multidrug resistance. Notably, microbes of these biofilms encoded biosynthetic gene clusters alongside ARGs, revealing complex ecological interactions and cross-domain crosstalk that shape microbial community dynamics [7]. 

Antarctica, often considered the last pristine continent, also harbours ARGs in various environments. ARG concentrations are notably higher around human settlements, such as research stations, compared to remote areas with minimal human activity. Studies in Antarctic wildlife and soils suggest that while anthropogenic ARGs are localised, historical ARGs likely reflect ancient horizontal gene transfer events and subsequent vertical inheritance. These findings underscore the potential of Antarctica as a model for studying the early evolution and transmission of ARGs in minimally impacted ecosystems [8, 9].

Similarly, some researchers have explored ancient permafrost from Arctic and Antarctic regions, uncovering antimicrobial resistance genes from pre-historic era. However, some of these approaches utilized targeted amplicon methods with primers designed for modern-day antimicrobial resistance genes, potentially leading to underestimation or overestimation of the true quantitative presence of antimicrobial resistance genes. Moreover, such approaches may potentially introduce PCR bias, obscuring the authentic nucleotide sequences of ancient antimicrobial resistance genes [3]. To address these challenges, we aimed to comprehensively explore ancient permafrost microbiomes and profile the entire resistome without employing any biased or targeted approaches. It is important to note that ancient antimicrobial resistance genes may not necessarily have representatives in present-day antibiotic resistance gene databases. These pristine environments offer undisturbed settings for studying microbial environments in the context of antimicrobial resistance with reduced post mortem DNA damage.

Earlier studies have suggested that antimicrobial resistance genes retrieved from ancient microbiomes are “divergent” from contemporary counterparts [10]. Building on these findings, we sought to address several key questions regarding ancient antimicrobial resistance genes. These include how divergent are antimicrobial resistance genes from palaeomicrobiomes from their modern counterparts, whether modern-day antimicrobial resistance genes, particularly after the introduction of antibiotics, have undergone positive evolutionary changes to become more effective against antibiotics, and whether there are differences in their collective modes of action. Additionally, we aimed to investigate whether bacteriophages generally carry antimicrobial resistance genes in palaeomicrobiological niches or if only a few of them harbour such genes due to selection pressure resulting from the irrational use of antibiotics in modern times.

Our study entails a comprehensive analysis of ancient microbiomes from existing genomic databases, profiling ARGs, identifying conserved domains relevant for antimicrobial resistance, and analysing selection pressure by estimating the Ka/Ks value. Furthermore, we aimed to meticulously classify potential complete bacteriophages and identify ARGs (if at all present) within them.

## Methods

### Data collection

Ancient metagenome datasets were systematically searched in the NCBI BioProject and community-curated SPAAM AncientMetagenomeDir using specific keywords to identify relevant studies. The keywords employed for the search included “Permafrost AND ancient Metagenome,” “Icecore and Metagenome,” and “Cryosoil AND metagenome.” Additionally, a taxonomy search with the taxon ID for permafrost metagenome ‘1082480’ was conducted.

The initial search yielded a total of 1427 Sequence Read Archives across 116 BioProjects upto June 2023. Subsequently, these projects underwent further scrutiny, specifically excluding targeted loci methods such as the 16 S amplicon method. The projects in which the antiquity of the sample was not clearly established—i.e., if it was not explicitly stated that the sample was obtained from a period predating the clinical introduction of antibiotics or was at least 70 years old—were also excluded. As a result, the final number of projects was narrowed down to a total of ten, namely PRJEB47746 [11], PRJNA266334 [12], PRJNA343018 [13], PRJNA350710 [14], PRJNA505516 [15], PRJNA596250 [16], PRJNA601698 [17], PRJNA680161 [18], PRJNA438924 [19], and PRJDB5557 [20]. The age of the samples in these projects ranged from 7000 years ago to 1100 kilo years ago (Supplementary Table S1).

### Geographical distribution of source data

**Fig. 1:**
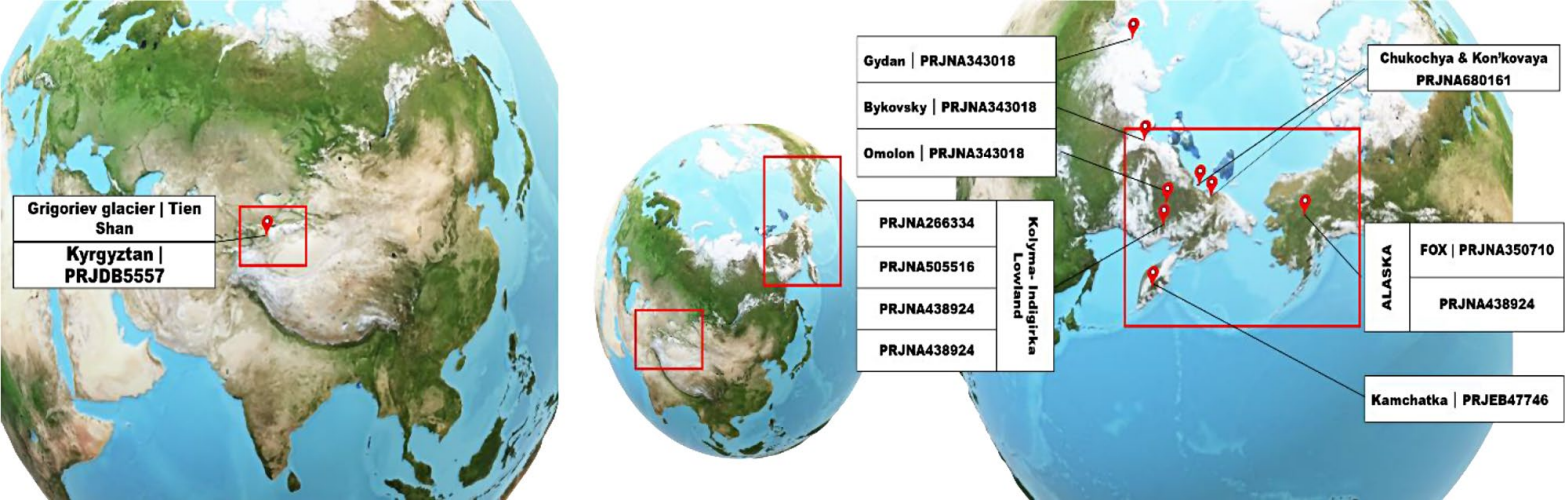
Geographical locations of studied samples across different projects

The above-mentioned studies were conducted in geographical locations primarily concentrated in Russia, with only three of them located outside Russia as shown in Fig. 1. These include two projects from Alaska, one focusing on Fox, renowned for cold regions research, and the other on the Grigoriev Glacier in the Tien Shan Mountain range of Kyrgyzstan, Central Asia. Other samples were obtained from diverse geographical locations within Russia, such as Kamchatka, a volcanic peninsula in the Russian Far East; the Kolyma-Indigirka Lowland, characterized by extensive permafrost in northeastern Siberia, Russia; regions such as Omolon, Gydan, and Bykovsky in Siberia, distinguished by their geological formations; specific sites along the Alazeya River within the Kolyma-Indigirka Lowland; geological formations like the Yedoma and Olyor suites within the same region; and areas encompassing the Alazeya River and Cape Chukochya. Additionally, Kon’kovaya which represent specific landmarks or formations within the Kolyma-Indigirka Lowland. The sampling methodologies employed in these studies follow a consistent approach aimed at minimising contamination during sample collection and processing. Dry drilling techniques were universally utilised, with no drilling fluid employed to avoid introducing contaminants into the permafrost samples. Core segments were extracted using air pressure or power drills, avoiding contamination from the surrounding environment. Additionally, in some cases, the outer peripheral layer of the cores was removed using a sterile knife to eliminate potential surface contaminants. The inner part of the cores, which is less likely to be affected by surface contamination, was targeted for sampling. Furthermore, in one study, the outer layer was coated with a control organism and then scraped off with a sterile knife to ensure thorough removal of any potential contaminants. DNA isolation has been carried out in clean room setups, with stringent measures to prevent cross-contamination.

Some projects, like PRJNA505516 and PRJNA680161, aimed to identify thriving microbes in ice cores using LIVE/DEAD staining. They distinguished the isolated genome from thriving microbes (based on LIVE/DEAD staining) as the intracellular genome, while the DNA from debris microbes was categorized as extracellular or environmental DNA (Supplementary Table S1). In PRJNA680161, replicates of samples have been prepared, with one set undergoing DNA damage repair using the preCR DNA repair cocktail, while the other set retained native DNA. Only the replicate with repaired DNA damage was selected for this study. In the project PRJNA438924, samples from both the Pleistocene and Holocene epochs were examined. However, only samples dating back to the Pleistocene were selected for inclusion in this study.

### Control metagenome data

As control samples, we selected additional studies alongside the three contemporary samples analysed in study PRJEB47746. One of these studies, categorised as ‘Pristine Contemporary,’ examined metagenomes sequenced from anoxic sites, including permafrost characterised by low water availability, low water activity, low temperature, restricted nutrient availability, and acidic conditions (PRJEB28336). Two studies, categorised as ‘Landfills,’ included metagenome samples from municipal waste dumps (PRJNA718480 and PRJNA606662). Additionally, we included metagenomes from agricultural lands in the USA, China, and Australia (PRJNA620778–PRJNA620785, PRJDB6359, and PRJNA317932), as well as metagenomes from arid lands in the same countries (PRJNA442912–PRJNA442920, PRJNA431914, and PRJNA647683), which were categorised under ‘Bareland.’ The respective resistomes for all these categories were profiled similarly.

### Profiling ancient ARGs in metagenomes

The paired-end FASTQ files were imported into the KBase^®^ server using the Import Paired-End Files utility [21]. Subsequently, the quality of the FASTQ files was assessed for the presence of adapters and read fragmentation using FASTQC tool. Adapters were removed using the Trimmomatic [22] tool, resulting in clean FASTQ files. These clean files were employed for metagenome assembly using the MEGAHIT tool in ‘meta large’ mode, with a minimum contig length threshold set at 1000 bps [23].

The assembled contigs were downloaded onto a Linux workstation, and the AMRFinderPlus tool was executed with a minimum coverage of 0.90 and minimum identity threshold of 0.40 [24]. Hits originating from partial gene fragments (PARTIALX) were filtered out. The nucleotide sequences of the remaining hits were then translated using the Transeq [25] command line tool. Next, the translated protein sequences underwent a search for conserved domains using InterPro [26].

For ARG count normalisation, the number of identifiable 5 S, 16 S, and 23 S genes from the assembled contigs was analysed using the Barrnap tool with default settings [27]. The absolute count of these genes was utilised to normalise the absolute count of ARGs obtained from the AMRFinderPlus run.

### Selection pressure estimation

The Ka/Ks ratio was computed for ORFs paired with their respective reference genes using Ka_Ks Calculator Tool version 3 [28]. Protein sequences underwent alignment using the MAFFT aligner [29]. This alignment data was utilized for codon alignment of nucleotide sequences employing PAL2NAL with the “-nogap” argument, aimed at removing gap lengths not divisible by 3 and non-overlapping regions [30]. Subsequently, the resulting clustal-formatted, codon-informed nucleotide alignment was transformed into an AXT file. This AXT file was then provided as input to the Ka_Ks Calculator, and the analysis was conducted using model-averaged method to estimate the selection pressure between the sequences.

### Taxonomic classification of ARG contigs

For rapid taxonomical identification of ARG bearing contigs, Kraken2 tool was employed to analyse the contigs against the Kraken2 Standard-8 microbial database (https://genome-idx.s3.amazonaws.com/kraken/k2_standard_08gb_20240112.tar.gz accessed on Feb 12, 2024) with default parameters [31]. The alpha diversity indices were then estimated using the Paleontological Software Tool (PAST v4) [32].

### Classification and retrieval of complete viral genomes

To classify viral contigs from the metagenome-assembled contigs, a hierarchical classification method was employed. Initially, the contigs were superficially classified using a deep learning method by DeepVirFinder, with a contig length cutoff of 3000 bp. Contigs with a viral score of at least 0.7 were selected for further classification [33]. Subsequently, the putative viral contigs underwent classification by VIBRANT version 1.2.1, which identifies viral hallmark proteins in the contigs [34]. Contigs classified as complete-circular by VIBRANT were then retrieved and subjected to AMRFinderPlus analysis to profile ARGs in the complete viruses with a minimum identity of 0.40 and a minimum coverage of 0.90.

## Results

### Profiling of ARGs in ancient microbiome

A total of approximately 2.3 tera basepairs of data were assembled into contigs using MEGAHIT, resulting in 13,919,982 contigs (> 1000 bps) in total. Three samples from the project PRJNA505516 and one sample from project PRJNA596250 did not yield any contigs (see Supplementary text, Table 1). Meanwhile, the samples from project PRJNA266334 yielded the lowest number of contigs. Among all the ancient metagenome contigs, AMRFinderPlus identified a total of 33,481 ARGs, while Barrnap version 0.7 identified 16,541 ribosomal RNA genes (5 S, 16 S, and 23 S). This translates to roughly 2 ARGs per ribosomal gene identified in the contigs. Notably, in one sample (PRJDB5557: DRR088405), 6 ARGs were identified, but no ribosomal genes could be detected. Table 1 illustrates that ARGs ranged as high as 3.25 ± 0.565 per rRNA gene in the project PRJNA47746, with the lowest occurrence observed in project PRJNA596250 (0.20 ± 0.028). In contrast, in contemporary samples from project PRJNA47746, the occurrence was 0.89; in PRJNA718480, it was 1.91; and in PRJNA606662, it was 0.44. The identified classes of resistance genes encompassed beta-lactam, tetracycline, macrolides including phenicols, fluoroquinolones, among others.

**Table 1:** Survey of ARGs present in metagenome of various ancient samples

Project accession	Total contigs count^a^	Total ARG count^b^	Total 5s, 16s, 23s count^c^	Mean RSU normalised ARG count ± SEM
PRJNA47746	83,36,212	24,851	9,613	3.25 ± 0.565
PRJNA266334	76	0	0	0
PRJNA680161	4,78,607	768	954	0.82 ± 0.065
PRJNA601698	6,43,806	1,166	1,356	1.23 ± 0.405
PRJNA350710	5,01,690	887	1,135	0.59 ± 0.153
PRJNA438924	11,99,868	4,140	1708	2.66 ± 0.359
PRJNA596250	90,014	56	273	0.20 ± 0.028
PRJNA505516	4,40,412	450	697	0.43 ± 0.113
PRJDB5557	42,788	141	60	1.13 ± 0.479
PRJNA343018	13,39,308	1,022	745	1.24 ± 0.228
Agriculture Numerous	62,68,032	11,897	785	2.48 ± 0.152
PRJDB6359	11,43,654	1345	2602	0.507 ± 0.066
PRJNA317932	43,05,038	8580	4263	1.748 ± 0.227
Bareland_Numerous	22,47,370	4251	4439	0.900 ± 0.098
PRJNA431914	29,32,493	5065	3472	1.460 ± 0.048
PRJNA647683	7,74,272	1231	1456	0.759 ± 0.117

a – Total number of contigs above 1000 bps length across different samples; b – Absolute number of ARGs in the assembled contigs with > 90% of coverage and > 0.40% of identity to the reference sequences in AMRFinder database; c – detected number of 5s, 16s, 23s ribosomal subunit genes detected by barrnap tool

### Distribution of identity percentages between ARGs from ancient metagenome and contemporary metagenome

**Fig. 2:**
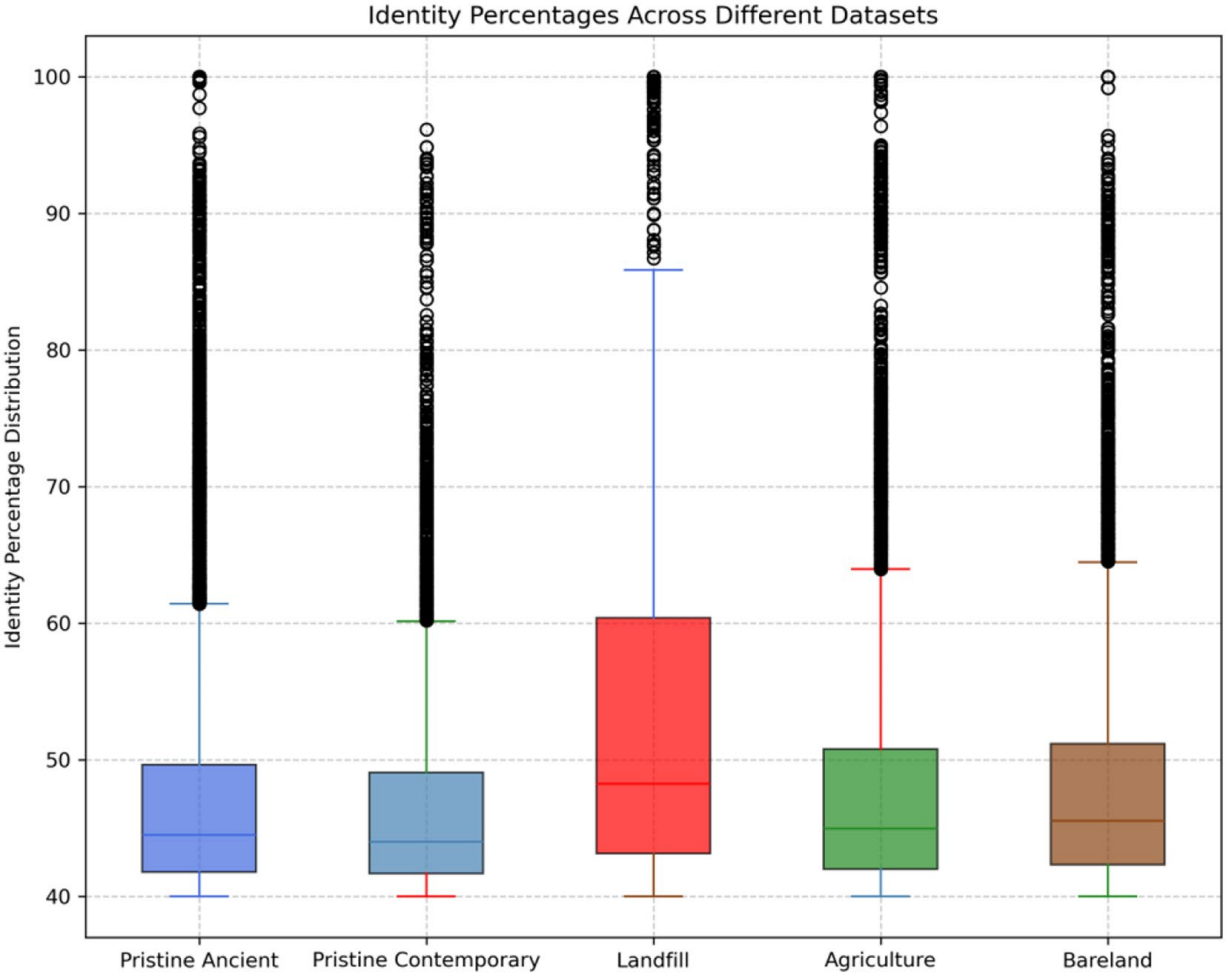
Box plot illustrating distribution of identity percentages between contemporary and ancient ARGs

The distribution of identity percentages was analysed, considering a minimum identity threshold of 40% and a relatively stringent minimum coverage of 90%. To verify the putative identification of genes as ARGs, a conserved domain search was conducted using Interpro. Across the samples and ages, the majority of the identified genes exhibited identity percentages between 40% and 60%, as depicted in Fig. 2. Notably, samples from project PRJEB47746 displayed a relatively well-distributed range of identity percentages for ARGs compared to their counterparts. Conversely, projects PRJNA505516 and PRJNA596250 exhibited only a few ARGs with identity percentages above 60%, coinciding with a relatively lower number of identified ARGs. In contemporary metagenome projects such as PRJNA606662 and PRJNA718480, the identity of ARGs was marginally higher than in the ancient samples. ARGs found in these samples demonstrated comfortable similarity percentages of up to 70% and 90%, respectively. In contrast, ARGs from contemporary pristine samples in project PRJEB28336 clustered within 60% similarity percentages. This suggests that metagenomic ARGs, regardless of age, exhibit very low similarity to present reference ARG database.

### Abundance of ARGs based on resistance mechanisms and classes

**Fig. 3:**
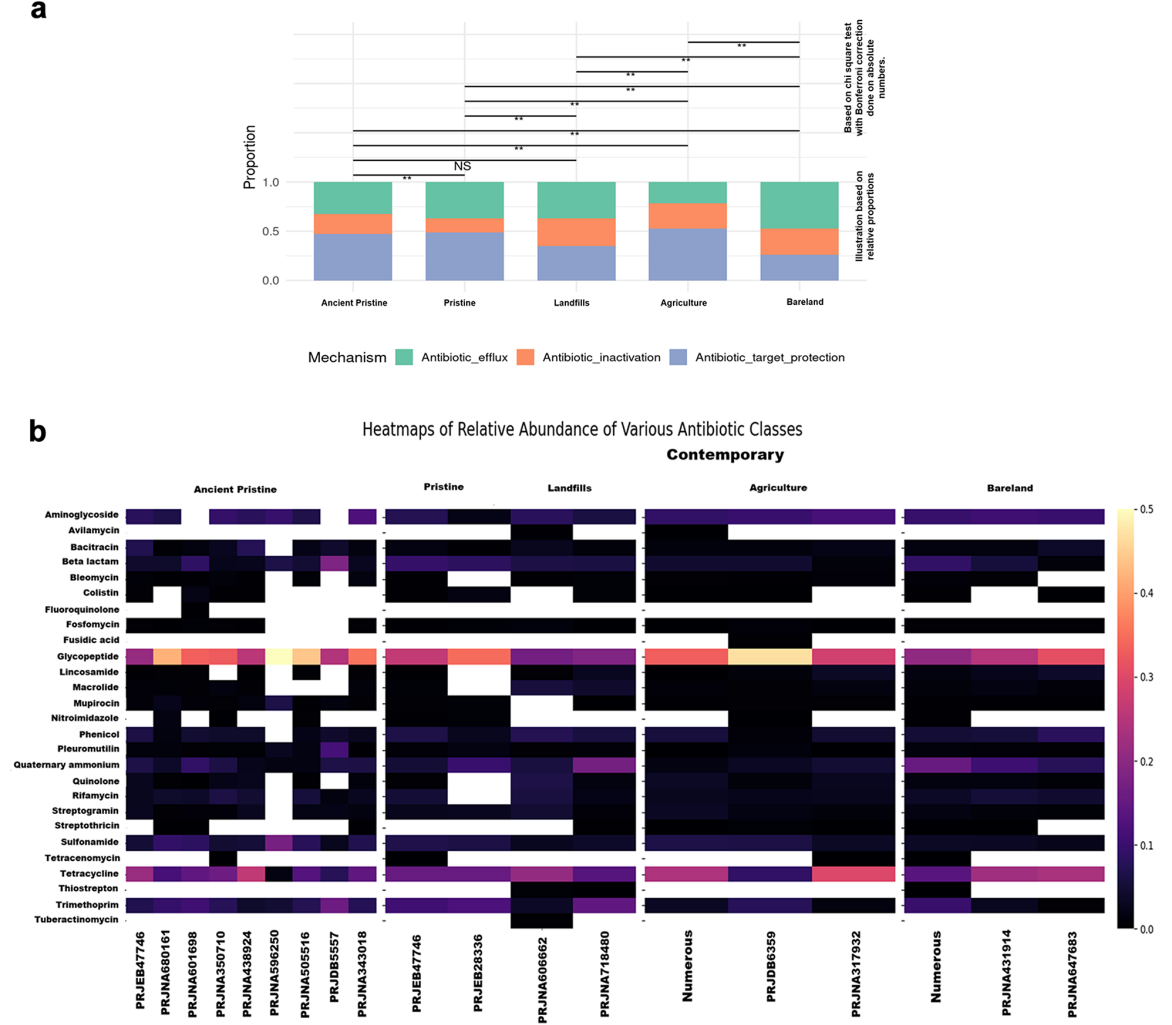
**a**: Bar plot illustrating the composition of ARGs belonging to Antibiotic inactivation, Antibiotic efflux and Antibiotic target protection/alteration/replacement in both ancient metagenomes and contemporary metagenomes. Chi square tests were conducted with absolute numbers and statistical significance was estimated with Bonferroni correction. **b**: Profiled ARGs classified based on resistance to different antibiotic classes. The Kruskal-Wallis test was conducted to assess the statistical significance of differences between antibiotic classes across each project pairwise. The results indicated no statistically significant differences between the antibiotic classes across the groups

The ARGs were classified into three classes based on their resistance mechanisms, such as antibiotic inactivation, antibiotic efflux, and antibiotic target protection/alteration/replacement as shown in Fig. 3. In the high-depth metagenomes from the project PRJEB47746, ARGs were almost equally distributed among these three resistance mechanisms. However, in projects PRJNA596250 and PRJNA680161, ARGs related to antibiotic target protection/alteration/replacement were notably dominant. Regarding contemporary samples, pristine metagenomes exhibited a higher proportion of ARGs associated with antibiotic target protection/alteration/replacement, while antibiotic inactivation ARGs were less prominent. Conversely, samples from landfills or municipal wastes showed insignificant differences between these three classes of resistance mechanisms.

**Fig. 4:**
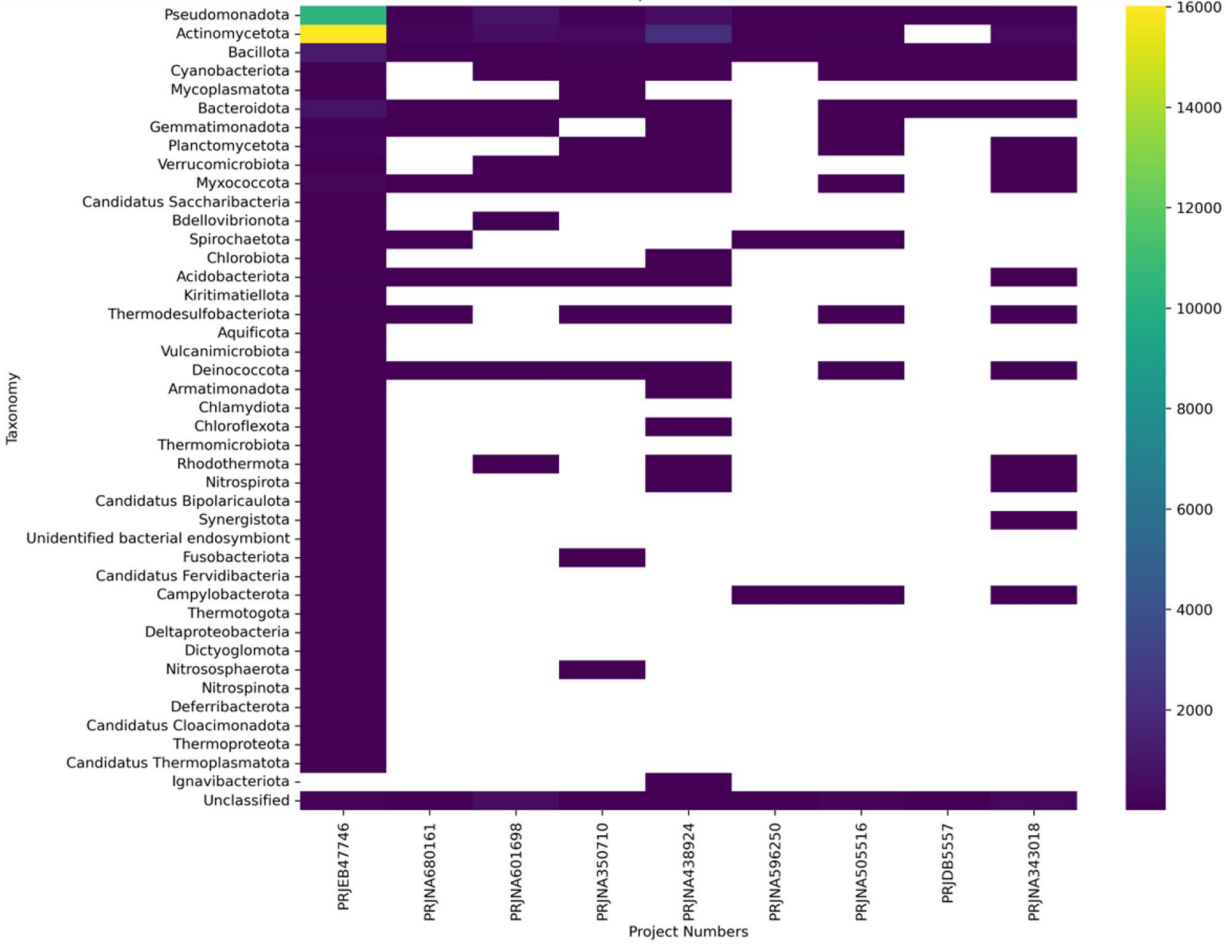
Heat map showing abundance of ARGs carrying phyla across different projects

In a similar manner, the profiled ARGs were categorized into classes of antibiotics to which they confer resistance, as illustrated in Fig. 4b. It can be observed that the ancient microbiome harbours resistance genes for Trimethoprim, quinolones, fosfomycin, mupirocin, nitroimidazole, sulfanamide, and other classes of antibiotics. Worth noting is that the mentioned classes of antibiotics are primarily synthetic or semi-synthetic. However, the genes conferring resistance are already ubiquitously present enzymes in bacteria, or resistance arises due to point mutations to the targets of antibiotics. For instance, resistance to Trimethoprim can occur through the inactivation of dihydrofolate reductase, while quinolone resistance may result from mutations in DNA gyrase and type IV topoisomerases. Dihydrofolate reductase, DNA gyrase and topoisomerase IV are all core essential enzymes in bacteria.

A heat map was generated based on the taxonomy assignments by the Kraken tool for contigs carrying ARGs, illustrating abundance versus taxa up to the phylum level (see Fig. 3). Among the studied projects, PRJEB47746 displayed a diverse range of taxa, with the majority belonging to *Pseudomonadota* and *Actinomycetota*. Interestingly, *Actinomycetota* did not exhibit the presence of ARGs in project PRJDB5557. Additionally, thermophilic phyla such as *Thermodesulfobacteriota*, *Deinococcota*, *Aquificota*, *Thermomicrobiota*, *Rhodothermota*, and *Thermoproteota* were not identified in this particular project. Following *Pseudomonadota* and *Actinomycetota*, *Bacillota* emerged as a consistent carrier of ARGs across all projects. Additionally, *Cyanobacteriota*, *Myxococcota*, *Bacteroidota*, *Gemmatimonadota*, *Acidobacteriota*, *Thermodesulfobacteriota*, and *Deinococcota* were identified as widespread carriers of ARGs across multiple projects.

### Factors affecting ARG abundance

We conducted a correlation analysis of the normalized ARG count with the age of samples, encompassing both contemporary and ancient samples (Supplementary Table S2). Using an inverse-ranked Spearman correlation analysis, we examined whether a decrease in age correlates with an increase in ARG count in the assembled contigs. The analysis revealed a mild negative correlation of -0.15, which was deemed statistically insignificant as it fell below the critical *t* value. Additionally, we explored whether diversity serves as a contributing factor to ARG abundance. Pearson’s correlation coefficient was employed to assess the correlation between the normalized ARG count and Simpson’s diversity coefficient. The analysis yielded a mild positive correlation of 0.17, which, like the previous correlation, was deemed statistically insignificant due to its smaller critical *t* value. Although we did not find any statistically significant correlation between the two tested factors, namely the diversity of taxa and the age of samples, it is possible that spatio-temporal factors and the presence of particular groups of organisms could be responsible for the varied distribution of ARGs across different metagenomes.

### Selection pressure on ancient versus contemporary antibiotic inactivation enzymes

The evolutionary pressure, measured by the Ka/Ks ratio on proteins, is often assessed using the ratio of substitution rates between non-synonymous and synonymous mutations. Initially developed to evaluate selection pressure in distantly divergent proteins, this method is now being applied to phylogenetically closer proteins and even proteins from single populations. In the case of antibiotic resistance genes (ARGs), enzymes capable of inactivating antibiotics, we aimed to determine the highest similarity percentage that would yield a divergence distance close to 1. Additionally, we sought to determine whether the popular Yang-Nielsen (YN) model or the more recent Model Averaged (MA) method in the Ka/Ks calculator would be optimal for identifying the best selection pressure ratio. To achieve this, we selected five pairs of genes ranging from 33 to 95% similarity and calculated their respective divergence distances and Ka/Ks ratios using both YN and MA methods. Notably, we included two 50% similarity sequence pairs, one from the same species and another from two different species. Our analysis revealed that the YN method could yield unrealistic divergence distances for ARGs and higher p-values compared to the MA method. Therefore, we concluded that the MA method was more suitable for our entire set of proteins. Additionally, we specifically chose proteins with similarity percentages equal to or less than 50% compared to the reference protein for Ka/Ks calculation Table 2.

**Table 2:** Validation of model selection for Ka/Ks estimation between ancient and contemporary ARGs

Species	AROs	Sequence similarity^*^	Model	Ka/Ks	*p* value	Divergence
*Achromobacter ruhlandii* vs. *Klebsiella pneumoniae*	OXA-925 vs. OXA-1	0.33	YN	0.212	4 × 10^− 18^	1.56
			MA	0.338	0	1
*Achromobacter ruhlandii* vs. *Ralstonia insidiosa*	OXA-925 vs. OXA-574	0.50	YN	0.105	1.38 × 10^− 38^	0.94
			MA	0.106	0	1
*Acinetobacter baumanii* vs. *Acinetobacter baumanii*	OXA-672 vs. OXA-366	0.50	YN	0.107	3 × 10^− 38^	1.08
			MA	0.122	0	1
*Acinetobacter parvuus* vs. *Acinetobacter gyllenbergii*	OXA-279 vs. OXA-671	0.75	YN	0.107	3.88 × 10^− 39^	0.54
			MA	0.107	1.86 × 10^− 108^	0.54
*Acinetobacter gyllenbergii* vs. *Acinetobacter gyllenbergii*	OXA-671 vs. OXA-672	0.95	YN	0.048	3.84 × 10^− 32^	0.14
			MA	0.059	3.81 × 10^− 33^	0.13

ARO- Antibiotic Resistance gene Ontology, YN – Yang Nielsen, MA- Model averaged method adapted by Ka_Ks calculator, * - percentage of similarity between two genes

**Fig. 5:**
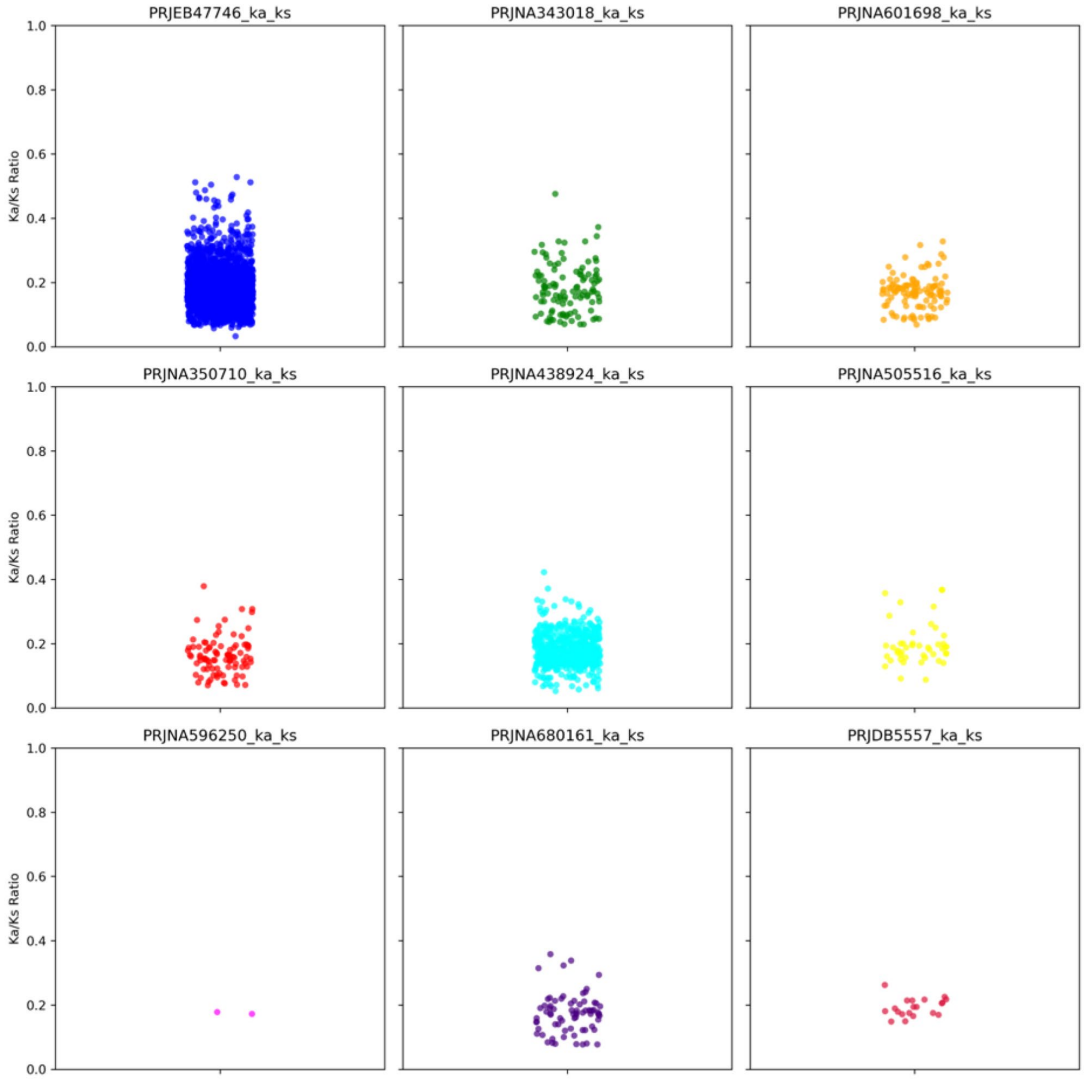
Distribution of Ka/Ks ratio for antibiotic inactivation genes from the ancient metagenome versus contemporary genes in the reference CARD database

All the tested antibiotic inactivation class ARGs were found to have Ka/Ks values well below 1, indicating that these genes experienced purifying selection. This suggests that the functions of these genes have been preserved, as expected for this class of genes. Notably, in project PRJEB47746, there was a diverse distribution of Ka/Ks ratios ranging from 0.01 to slightly above 0.5, as shown in Fig. 5. Across all projects, antibiotic inactivation ARGs exhibited Ka/Ks values concentrated around 0.2, indicating strong negative selection pressure on these ARGs, as assessed by the MA method in the Ka/Ks calculator.

### Complete viral genome retrieval and ARG profiling

In order to retrieve viral contigs from the metagenome and assess the presence of antimicrobial resistance genes (ARGs) within them, a systematic approach was adopted. Initially, a hierarchical classification method was utilized to classify the contigs assembled from the metagenome. DeepVirFinder, employing a deep learning method, was first employed for a preliminary classification of the contigs, with a length cutoff set at 3000 bp. Contigs scoring a viral probability of at least 0.7 were further selected for classification. Subsequently, VIBRANT version 1.2.1 was employed for a more detailed classification, focusing on the identification of viral hallmark proteins within the contigs. Contigs classified as complete-circular by VIBRANT were then extracted and subjected to AMRFinderPlus analysis to profile ARGs within the complete viruses, employing a minimum identity and coverage threshold of 0.40 each. Through this comprehensive analysis, a total of 380 putative complete viruses were identified from seven projects (Table 3) and none of these viruses were found to harbour ARGs.

**Table 3:** Complete viral sequences identified by VIBRANT

Project Number	Number of complete viral genomes	Smallest genome size	Largest genome size	Average genome size
PRJEB47746	282	3139	175,833	31,103
PRJNA680161	13	3940	103,189	49,413
PRJNA350710	16	3996	7581	5487.1
PRJNA601698	19	3053	69,374	20162.4
PRJNA343018	6	5513	5513	5513
PRJNA438924	38	3322	114,447	43895.2
PRJNA505516	6	5513	42,098	11610.5

## Discussion

Our study encompassed a total of 2.3 tera base pairs of data, either assembled or directly profiled for ARG prevalence using prominent tools and databases such as AMRFinderPlus, KMA, and the CARD and RefGene catalogues of Antibiotic Resistance Genes. Approximately 1.3 billion contigs, each exceeding 1000 bps, were generated through the assembly process. However, four sets of raw metagenome reads from two distinct projects could not be assembled into contigs.

The AMRFinder analysis identified about 33,000 ARGs across all projects, meeting the criteria of at least 0.90 coverage and 0.40 identity with the reference database. To ensure accurate selection pressure analysis downstream, a coverage threshold of 0.90 was set, allowing only BLASTX results to remain while genes identified as putative ARGs by PARTIALX were removed. Additionally, ARGs with internal stop codons were included. Previous research highlighted the prevalence of divergent ARGs with low identity thresholds, potentially indicating phenotypic antimicrobial resistance characteristics [10]. To mitigate false positives, the presence of conserved domains in putative ARGs was verified using InterPro search.

The relative abundance of ARGs in ancient microbiomes was assessed by profiling ubiquitously present ribosomal genes. It was found that there were at least 2 ARGs for every identified rRNA gene (5s, 16s, and 23s collectively) in the ancient metagenome. Notably, the relativistic estimate varied greatly among different projects including contemporary studies.

The distribution of identity percentages of ARGs from all samples was plotted into a box and whiskers plot, revealing a prevalence of ARGs around 40% similarity regardless of the microbiome’s temporal status. However, municipal waste and landfill metagenomes exhibited a higher likelihood of finding ARGs above 0.70 compared to pristine/agriculture/bareland counterparts. It is noteworthy that the majority of ARGs were identified below 60% identity in both the ancient microbiomes and the contemporary microbiomes. But just mere detection of ARGs does not mean they would be expressed phenotypically, it can only be verified experimentally.

The classification of ARGs into broad categories—antibiotic inactivation, antibiotic efflux, or antibiotic target alteration/protection/replacement—was enabled by the CARD Resistance Gene Identifier tool. Interestingly, while ancient microbiomes showed fewer inactivation-type ARGs, contemporary metagenomes displayed a more even distribution among the three classifications. Statistically, at the mechanism level, the functional pressures exerted by different environments (e.g., agriculture, bareland) drive significant differences. However, these pressures might not target individual antibiotic classes strongly enough to cause significant differences at that level. This may suggest that environmental pressures act on broader functional traits rather than specific antibiotic resistances.

Further investigation aimed to understand the correlation between ARG prevalence and factors such as age and diversity. Initially, we investigated whether age influences the occurrence of ARGs. Utilizing an inverse-ranked Spearman correlation analysis, we found that age had an insignificant effect on ARG prevalence. Subsequently, we examined whether diversity correlates with ARG presence. Taxonomy was assigned to contigs using the Kraken2 tool, and Simpson diversity indices were estimated. Pearson correlation coefficients were then calculated between diversity indices and ARG abundance. This analysis also revealed an insignificant correlation between ARGs and the diversity of organisms present. In project PRJEB47746, a diverse range of contigs were found to carry ARGs, whereas in project PRJDB5557, only a few specific taxa were associated with ARGs.

Interestingly, thermophiles were present in all projects except PRJDB5557. These metagenomes, sourced from the Chuckochi region, likely experienced volcanic activity around 10 million years ago, potentially leading to the presence of active hot springs long after the landmass formation. Consequently, taxa such as *Aquificae*, *Thermomicrobiota*, and *Deinococcota*, which thrive in thermophilic environments, were identified. In contrast, PRJDB5557 samples were collected from the Tien Shan Mountain range, formed by the collision of Eurasian tectonic plates.

For our selection pressure estimation, we focused solely on antibiotic inactivation-type ARGs. This decision was made because even single nucleotide polymorphisms (SNPs) can affect the functionality of the resistance genes in the other two categories, which do not require substantial evolutionary modifications. We calculated the ratio of non-synonymous mutations to synonymous mutations and found that all the antibiotic inactivation ARGs exhibited purifying selection. It is not surprising that the functionality of such enzymes is under negative selection pressure. Additionally, the timeframe tested here is relatively short in comparison to the evolutionary scale of microorganisms, if we analyse further ancient sequence there is a possibility of finding positive selection pressure. This finding is consistent with other studies reporting purifying selection in ARGs overall [35]. However, it’s important to note that many studies estimate selection pressure between closely related genes or those with considerably higher similarity, which can result in confounding estimates of selection pressure. Similar sequences typically exhibit lower substitution rates, making estimates derived from such low rates statistically unreliable [35, 36]. Thus, we chose 50% identity as a cutoff below which the divergence would be sufficient to enable ka/ks analysis between them.

In our study, for samples that could not be assembled into contigs, we directly mapped the adapter-removed reads to the ARG database using the KMA aligner. However, even with this approach, we did not identify any ARGs in these samples. Additionally, it appears challenging to identify any genes from these reads, similar to the observation with small subunit ribosomal genes (see Supplementary text, Table 1). Interestingly, two of these samples (labelled ‘i’ in superscript) possibly harboured thriving organisms according to the original study.

Notably, from the contigs classified by VIBRANT from the metagenome-assembled contigs, no ARGs were found above the set threshold. Several reports claim that bacteriophages carry ARGs, while few report contrary findings. According to our literature review, we believe that bacteriophages typically do not carry ARGs, but they do so rarely [37]. Additionally, we learned that some specialized bacteriophages, known as phage-plasmids, can harbour ARGs. We sought to understand the scenario ages ago regarding bacteriophages retrieved from ancient permafrost. However, based on the limited results obtained in our study, it is too early to conclude whether ARGs are being carried in a few phages due to pressure from antibiotic usage in the modern times since there are no ARGs identified in the putative viral genomes from the ancient microbiome.

Based on these findings, it was concluded that the distribution of identity percentages between contemporary and ancient metagenome-sourced ARGs remained similar. Additionally, antibiotic inactivation-type ARGs from ancient microbiomes did not seem to have undergone substantial evolutionary changes to become more effective against antimicrobials in the antibiotic era. Importantly, none of the identified putative viruses carried ARGs in ancient times, although further investigation is warranted to see if the presence of few ARGs in the contemporary phage genomes are due to selection pressure from irrational use of antibiotics.

## Conclusion

The popular maxim “Everything is everywhere, but environment selects” appears to hold true to some extent concerning antibiotic resistance genes. Antibiotic resistance has always existed and will continue to do so. However, it is known that anthropogenic influences have significantly exacerbated the effects of antibiotic resistance. Although these genes have been subject to negative evolutionary selection, they may exhibit greater phenotypic expression in contemporary times compared to the pre-antibiotic era.

## Electronic supplementary material

Below is the link to the electronic supplementary material.

Supplementary Material 1

Supplementary Material 2

Supplementary Material 3

## Data Availability

All the metagenomic raw data were retrieved from NCBI SRA with the following accessions: PRJEB47746, PRJNA266334, PRJNA343018, PRJNA350710, PRJNA505516, PRJNA596250, PRJNA601698, PRJNA680161, PRJNA438924, PRJDB5557, PRJEB28336, PRJNA718480 and PRJNA606662. All the scripts used during the study are available at the following GitHub repository - https://github.com/GomathiNayagam/ARGs_Ancient_permafrost.

## Abbreviations

ARG: Antibiotic resistance gene
ARO: Antibiotic Resistance gene Ontology
YN: Yang-Nielsen
MA: Model averaged method

## References

[1] Chen S-C, Sun G-X, Yan Y, Konstantinidis KT, Zhang S-Y, Deng Y, et al. The great oxidation event expanded the genetic repertoire of arsenic metabolism and cycling. Proc Natl Acad Sci USA. 2020. 10.1073/pnas.2001063117. 32350143 10.1073/pnas.2001063117 PMC7229686

[2] Khademian M, Imlay JA. How microbes evolved to Tolerate Oxygen. Trends Microbiol. 2021. 10.1016/j.tim.2020.10.001. 33109411 10.1016/j.tim.2020.10.001 PMC8043972

[3] D’Costa VM, King CE, Kalan L, Morar M, Sung WWL, Schwarz C, et al. Antibiotic resistance is ancient. Nature. 2011. 10.1038/nature10388. 21881561 10.1038/nature10388

[4] Spagnolo F, Trujillo M, Dennehy JJ. Why Do Antibiotics Exist? Lopatkin, Barnard College A, Yount J, editors. *mBio*. 2021; 10.1128/mBio.01966-21 10.1128/mBio.01966-21 PMC8649755 34872345

[5] Sankaranarayanan G, Kodiveri Muthukaliannan G. Exploring antimicrobial resistance determinants in the Neanderthal microbiome. Kumar A, editor. *Microbiol Spectr*. 2024; 10.1128/spectrum.02662-23 10.1128/spectrum.02662-23 PMC11302244 38916350

[6] Gattinger D, Schlenz V, Weil T, Sattler B. From remote to urbanized: dispersal of antibiotic-resistant bacteria under the aspect of anthropogenic influence. Sci Total Environ. 2024. 10.1016/j.scitotenv.2024.171532. 38458439 10.1016/j.scitotenv.2024.171532

[7] Busi SB, De Nies L, Pramateftaki P, Bourquin M, Kohler TJ, Ezzat L et al. JA Gralnick editor 2023 Glacier-Fed Stream Biofilms Harbor Diverse resistomes and Biosynthetic Gene clusters. Microbiol Spectr 10.1128/spectrum.04069-22. 10.1128/spectrum.04069-22 PMC9927545 36688698

[8] Hwengwere K, Paramel Nair H, Hughes KA, Peck LS, Clark MS, Walker CA. Antimicrobial resistance in Antarctica: is it still a pristine environment? *Microbiome*. 2022; 10.1186/s40168-022-01250-x 10.1186/s40168-022-01250-x PMC9072757 35524279

[9] Van Goethem MW, Pierneef R, Bezuidt OKI, Van De Peer Y, Cowan DA, Makhalanyane TP. A reservoir of ‘historical’ antibiotic resistance genes in remote pristine Antarctic soils. Microbiome. 2018. 10.1186/s40168-018-0424-5. 29471872 10.1186/s40168-018-0424-5 PMC5824556

[10] Rascovan N, Telke A, Raoult D, Rolain JM, Desnues C. Exploring divergent antibiotic resistance genes in ancient metagenomes and discovery of a novel beta-lactamase family. Environ Microbiol Rep. 2016. 10.1111/1758-2229.12453. 27518706 10.1111/1758-2229.12453

[11] Rigou S, Christo-Foroux E, Santini S, Goncharov A, Strauss J, Grosse G, et al. Metagenomic survey of the microbiome of ancient siberian permafrost and modern Kamchatkan cryosols. microLife. 2022. 10.1093/femsml/uqac003. 37223356 10.1093/femsml/uqac003 PMC10117733

[12] Krivushin K, Kondrashov F, Shmakova L, Tutukina M, Petrovskaya L, Rivkina E. Two metagenomes from late pleistocene northeast siberian permafrost. Genome Announc. 2015. 10.1128/genomeA.01380-14. 25555741 10.1128/genomeA.01380-14 PMC4293628

[13] Vishnivetskaya T, Spirina E, Shmakova L, Tutukina M, Li Z, Wu X, et al. Metagenomes from late pleistocene ice complex sediments of the siberian Arctic. Stewart FJ, editor. Microbiol Resour Announc. 2019. 10.1128/MRA.01010-19. 31649080 10.1128/MRA.01010-19 PMC6813392

[14] Mackelprang R, Burkert A, Haw M, Mahendrarajah T, Conaway CH, Douglas TA, et al. Microbial survival strategies in ancient permafrost: insights from metagenomics. ISME J. 2017. 10.1038/ismej.2017.93. 28696425 10.1038/ismej.2017.93 PMC5607373

[15] Liang R, Lau M, Vishnivetskaya T, Lloyd KG, Wang W, Wiggins J et al. Predominance of Anaerobic, Spore-Forming Bacteria in Metabolically Active Microbial Communities from Ancient Siberian Permafrost. Stams AJM, editor. *Appl Environ Microbiol*. 2019; 10.1128/AEM.00560-19 10.1128/AEM.00560-19 PMC6643238 31152014

[16] Sipes K, Almatari A, Eddie A, Williams D, Spirina E, Rivkina E, et al. Eight metagenome-assembled genomes provide evidence for Microbial Adaptation in 20,000- to 1,000,000-Year-old siberian permafrost. Kelly RM, editor. Appl Environ Microbiol. 2021. 10.1128/AEM.00972-21. 34288700 10.1128/AEM.00972-21 PMC8432575

[17] Wu X, Almatari AL, Cyr WA, Williams DE, Pfiffner SM, Rivkina EM, et al. Microbial life in 25-m-deep boreholes in ancient permafrost illuminated by metagenomics. Environ Microbiome. 2023. 10.1186/s40793-023-00487-9. 37055869 10.1186/s40793-023-00487-9 PMC10103415

[18] Liang R, Li Z, Lau Vetter MCY, Vishnivetskaya TA, Zanina OG, Lloyd KG, et al. Genomic reconstruction of fossil and living microorganisms in ancient siberian permafrost. Microbiome. 2021. 10.1186/s40168-021-01057-2. 34001281 10.1186/s40168-021-01057-2 PMC8130349

[19] Barbato RA, Jones RM, Douglas TA, Esdale J, Foley K, Perkins EJ, et al. Alaskan palaeosols in modern times: deciphering unique microbial diversity within the late-holocene. Holocene. 2022. 10.1177/09596836221101249.

[20] Segawa T, Takeuchi N, Fujita K, Aizen VB, Willerslev E, Yonezawa T. Demographic analysis of cyanobacteria based on the mutation rates estimated from an ancient ice core. Heredity. 2018. 10.1038/s41437-017-0040-3. 29302050 10.1038/s41437-017-0040-3 PMC5943335

[21] Arkin AP, Cottingham RW, Henry CS, Harris NL, Stevens RL, Maslov S, et al. KBase: the United States Department of Energy Systems Biology Knowledgebase. Nat Biotechnol. 2018. 10.1038/nbt.4163. 29979655 10.1038/nbt.4163 PMC6870991

[22] Bolger AM, Lohse M, Usadel B. Trimmomatic: a flexible trimmer for Illumina sequence data. Bioinformatics. 2014. 10.1093/bioinformatics/btu170. 24695404 10.1093/bioinformatics/btu170 PMC4103590

[23] Li D, Liu C-M, Luo R, Sadakane K, Lam T-W. MEGAHIT: an ultra-fast single-node solution for large and complex metagenomics assembly via succinct *de bruijn* graph. Bioinformatics. 2015. 10.1093/bioinformatics/btv033. 25609793 10.1093/bioinformatics/btv033

[24] Feldgarden M, Brover V, Gonzalez-Escalona N, Frye JG, Haendiges J, Haft DH, et al. AMRFinderPlus and the reference gene catalog facilitate examination of the genomic links among antimicrobial resistance, stress response, and virulence. Sci Rep. 2021. 10.1038/s41598-021-91456-0. 34135355 10.1038/s41598-021-91456-0 PMC8208984

[25] Madeira F, Pearce M, Tivey ARN, Basutkar P, Lee J, Edbali O, et al. Search and sequence analysis tools services from EMBL-EBI in 2022. Nucleic Acids Res. 2022. 10.1093/nar/gkac240. 35412617 10.1093/nar/gkac240 PMC9252731

[26] Paysan-Lafosse T, Blum M, Chuguransky S, Grego T, Pinto BL, Salazar GA, et al. InterPro in 2022. Nucleic Acids Res. 2023. 10.1093/nar/gkac993. 36350672 10.1093/nar/gkac993 PMC9825450

[27] Torsten S, Seemann T. 2013. Barrnap 0.7: rapid ribosomal RNA prediction.

[28] Zhang Z, Li J, Zhao X-Q, Wang J, Wong GK-S, Yu J, KaKs_Calculator. Calculating Ka and Ks through Model Selection and Model Averaging. Genom Proteom Bioinform. 2006. 10.1016/S1672-0229(07)60007-2. 10.1016/S1672-0229(07)60007-2 PMC5054075 17531802

[29] Katoh K. MAFFT: a novel method for rapid multiple sequence alignment based on fast Fourier transform. Nucleic Acids Res. 2002. 10.1093/nar/gkf436. 12136088 10.1093/nar/gkf436 PMC135756

[30] Suyama M, Torrents D, Bork P. PAL2NAL: robust conversion of protein sequence alignments into the corresponding codon alignments. Nucleic Acids Res. 2006. 10.1093/nar/gkl315. 16845082 10.1093/nar/gkl315 PMC1538804

[31] Wood DE, Lu J, Langmead B. Improved metagenomic analysis with Kraken 2. Genome Biol. 2019. 10.1186/s13059-019-1891-0. 31779668 10.1186/s13059-019-1891-0 PMC6883579

[32] Hammer Ø, Harper DA, Ryan. PAST: Paleontological Statistics Software Package for Education and Data Analysis. Palaeontol Electron. 2001;4:9.

[33] Ren J, Song K, Deng C, Ahlgren NA, Fuhrman JA, Li Y, et al. Identifying viruses from metagenomic data using deep learning. Quant Biol. 2020. 10.1007/s40484-019-0187-4. 34084563 10.1007/s40484-019-0187-4 PMC8172088

[34] Kieft K, Zhou Z, Anantharaman K. VIBRANT: automated recovery, annotation and curation of microbial viruses, and evaluation of viral community function from genomic sequences. Microbiome. 2020. 10.1186/s40168-020-00867-0. 32522236 10.1186/s40168-020-00867-0 PMC7288430

[35] Debroas D, Siguret C. Viruses as key reservoirs of antibiotic resistance genes in the environment. ISME J. 2019. 10.1038/s41396-019-0478-9. 31358910 10.1038/s41396-019-0478-9 PMC6794266

[36] Kryazhimskiy S, Plotkin JB. The Population Genetics of dN/dS. Gojobori T, editor. *PLoS Genet*. 2008; 10.1371/journal.pgen.1000304 10.1371/journal.pgen.1000304 PMC2596312 19081788

[37] Gomathinayagam S, Kodiveri Muthukaliannan G. Dynamics of antibiotic resistance genes in plasmids and bacteriophages. Crit Rev Microbiol. 2024. 10.1080/1040841X.2024.2339262. 38651513 10.1080/1040841X.2024.2339262

