# Supplementary material for: Millennial-scale microbiome analysis reveals ancient antimicrobial resistance conserved despite modern selection pressures
Source: Environ Microbiome. 2024 Dec 18;19:110. doi: 10.1186/s40793-024-00652-8 (PMC11657988; doi:10.1186/s40793-024-00652-8)
Supplement: Supplementary file 1 — Supplementary Material 1 [file 40793_2024_652_MOESM1_ESM.docx]

Supplementary text for “Millennial-Scale Microbiome Analysis Reveals Ancient Antimicrobial Resistance Conserved Despite Modern Selection Pressures”

Mapping based assembly

For samples that did not yield any assembled contigs, a k-mer-based read-level ARG finding approach was implemented using KMA tools ^1^. Initially, the CARD-ARG database and NCBI's Refgene catalogue for Antibiotic Resistance Genes (v2020.10.12) were merged ^2^, and a KMA index was generated. Subsequently, the KMA aligner was employed to align the reads to this index, and the output was rendered as a SAM format file. Similarly, the GTDB SSU (bac120 release 214) database was indexed, and the reads were aligned to this index to identify SSU (Small Subunit) genes for ARG count normalization ^3^. A mapping coverage threshold of 0.70 was selected for both ARGs and SSUs from the SAM files.

Insights from mapping first assembly attempt

ARGs were identified from samples that did not yield any or had a low number of contigs, which were then subjected to mapping against reference ARG databases using the KMA aligner. No ARGs above the set threshold coverage of 70% were identified in these samples. In contrast, a total of 15 ribosomal proteins were identified above the set threshold coverage in all samples combined. Sample SRR13615822 did not yield any ARGs or ribosomal proteins due to low depth of coverage. Similarly, sample SRR8188256 did not yield any of these genes despite having substantial depth. Notably, this sample was derived from environmental DNA. Two other samples, SRR8188252 and SRR8188255, which likely originated from intracellular bacterial cells, had 8 and 1 ribosomal proteins, respectively, but no ARGs.

Table 1: ARGs and ribosomal proteins identified directly from the raw reads by KMA

| Project | Accession no. | Base pairs | ARG above 0.70 | SSU above 0.70 |
| --- | --- | --- | --- | --- |
| PRJNA266334 | SRR1653578 | 91 Mbps | 0 | 5 |
|  | SRR1653579 | 91 Mbps | 0 | 1 |
| PRJNA505516 | SRR8188252^i^ | 7.1 Gbps | 0 | 8 |
|  | SRR8188255^i^ | 6 Gbps | 0 | 1 |
|  | SRR8188256^e^ | 2.8 Gbps | 0 | 0 |
| PRJNA596250 | SRR13615822 | 5 Kbps | 0 | 0 |

i- DNA extracted intracellularly from probably living bacteria. e- environmental DNA from the ancient permafrost;

References:

1. Clausen, P. T. L. C., Aarestrup, F. M. & Lund, O. Rapid and precise alignment of raw reads against redundant databases with KMA. *BMC Bioinformatics* **19**, 307 (2018).

2. Alcock, B. P. *et al.* CARD 2020: antibiotic resistome surveillance with the comprehensive antibiotic resistance database. *Nucleic Acids Res.* gkz935 (2019) doi:10.1093/nar/gkz935.

3. GTDB all SSU database July 2023 release.
